# Modern Cremation

**Published:** 1900-03

**Authors:** 


**Modern Cremation.** By Sir H. THOMPSON, Bart., M.B. Third Edition. Pp. xii., 187. London: Smith, Elder, & Co. 1899.

The contents of this book are sufficiently indicated by its sub-title, "Cremation: Its History and Practice to the present date, with information relating to all recently improved arrangements made by the Cremation Society of England." Sir Henry Thompson undoubtedly makes out a strong case in favour of cremation, but we doubt whether public opinion is yet sufficiently advanced to permit its general adoption. We learn that from 1885 to 1898 inclusive a total of 1,283 cremations took place, of which the largest number in any year, 240, were performed in 1898.

